# The Signal Sequence Coding Region Promotes Nuclear Export of mRNA

**DOI:** 10.1371/journal.pbio.0050322

**Published:** 2007-12-04

**Authors:** Alexander F Palazzo, Michael Springer, Yoko Shibata, Chung-Sheng Lee, Anusha P Dias, Tom A Rapoport

**Affiliations:** 1 Department of Cell Biology, Harvard Medical School, Boston, Massachusetts, United States of America; 2 Department of Systems Biology, Harvard Medical School, Boston, Massachusetts, United States of America; University of California San Francisco, United States of America

## Abstract

In eukaryotic cells, most mRNAs are exported from the nucleus by the transcription export (TREX) complex, which is loaded onto mRNAs after their splicing and capping. We have studied in mammalian cells the nuclear export of mRNAs that code for secretory proteins, which are targeted to the endoplasmic reticulum membrane by hydrophobic signal sequences. The mRNAs were injected into the nucleus or synthesized from injected or transfected DNA, and their export was followed by fluorescent in situ hybridization. We made the surprising observation that the signal sequence coding region (SSCR) can serve as a nuclear export signal of an mRNA that lacks an intron or functional cap. Even the export of an intron-containing natural mRNA was enhanced by its SSCR. Like conventional export, the SSCR-dependent pathway required the factor TAP, but depletion of the TREX components had only moderate effects. The SSCR export signal appears to be characterized in vertebrates by a low content of adenines, as demonstrated by genome-wide sequence analysis and by the inhibitory effect of silent adenine mutations in SSCRs. The discovery of an SSCR-mediated pathway explains the previously noted amino acid bias in signal sequences and suggests a link between nuclear export and membrane targeting of mRNAs.

## Author Summary

In eukaryotic cells, precursors of messenger RNAs (mRNAs) are synthesized and processed in the nucleus. During processing, noncoding introns are spliced out, and a cap and poly-adenosine sequence are added to the beginning and end of the transcript, respectively. The resulting mature mRNA is exported from the nucleus to the cytoplasm by crossing the nuclear pore. Both the introns and the cap help to recruit factors that are necessary for nuclear export of an mRNA. Here we provide evidence for a novel mRNA export pathway that is specific for transcripts coding for secretory proteins. These proteins contain signal sequences that target them for translocation across the endoplasmic reticulum membrane. We made the surprising observation that the signal sequence coding region (SSCR) can serve as a nuclear export signal of an mRNA that lacks an intron or functional cap. Even the export of an intron-containing natural mRNA was enhanced by its SSCR. The SSCR export signal appears to be characterized in vertebrates by a low content of adenines. Our discovery of an SSCR-mediated pathway explains the previously noted amino acid bias in signal sequences, and suggests a link between nuclear export and membrane targeting of mRNAs.

## Abstract 3

Signal sequences, which target newly synthesized secretory proteins to the endoplasmic reticulum, are encoded by adenine-depleted nucleotide sequences that promote the nuclear export of the mRNAs.

## Introduction

In eukaryotes, mRNAs are synthesized and processed in the nucleus before they are transported through the nuclear pores into the cytoplasm, where they are translated into proteins. Nuclear export of most mRNAs is mediated by the conserved transcription export (TREX) complex that is comprised of the Tho complex, UAP56, and Aly. In vertebrates, the TREX components are recruited to the 5′ end of newly synthesized transcripts by the combined action of the 5′ cap binding complex, CBP80/20, and factors that are loaded during the splicing of the intron closest to the 5′ cap [1–4]. Once assembled, the TREX complex recruits the heterodimer TAP/p15 as an export factor [5,6]. TAP interacts with nucleoporins directly [7–9] or through the factor Rae1 [10,11] and may thus allow bound transcripts to enter and eventually pass through the nuclear pores. It remains unclear how mRNAs exit the pores on the cytoplasmic side, but RNA helicases, such as Dbp5, may be involved [12,13]. Although many details of the export mechanism remain to be clarified, it seems clear that the efficient export of most mRNAs requires both splicing and a functional cap.

Not all mRNAs follow this canonical export pathway. In higher eukaryotes, transcripts coding for cyclin D [14] and other regulators of cell division [15] use elements in the 3′ untranslated region (UTR) as well as the cap binding protein eIF4E to engage the exportin protein Crm1. A Crm1-dependent pathway is also used for the export of the intron-containing RNA genome of the human immunodeficiency virus (HIV) [16]. In macrophages, the export of interferon-induced transcripts is sensitive to the levels of Nup96, a component of the nuclear pore, whereas other transcripts are insensitive [17]. In Saccharomyces cerevisiae, the export of some mRNAs requires either the Aly ortholog Yra1p or the TAP ortholog Mex67p, but not both [18]. Transcripts coding for heat-shock proteins, such as Hsp70p, are also exported by an Aly-independent pathway that is stimulated under stress conditions [19]. Together, these data indicate that there may be distinct export pathways for certain classes of mRNAs, which may be related to the different functions of the final translation products.

One specialized class of mRNAs codes for secretory proteins. These mRNAs are often translated by ribosomes that are targeted to the endoplasmic reticulum (ER) membrane. As a result, the mRNAs also become membrane-bound. In addition, it is possible that these mRNAs associate with the ER membrane in a ribosome-independent manner by interacting with RNA-binding proteins [20–22]. These proteins may be loaded onto the mRNAs in the nucleus, during their passage through the nuclear pores, or in the cytoplasm. Thus, the membrane localization of these mRNAs may require factors that mediate nuclear export and distribution in the cytoplasm, which are distinct from factors used by mRNAs translated on free ribosomes in the cytoplasm.

A major characteristic of a secretory protein is a hydrophobic signal sequence close to its N terminus. The signal sequence is first recognized by the signal-recognition particle (SRP) as it emerges from the translating ribosome and is then transferred into the protein-conducting channel formed by the Sec61p complex [23–25]. In most cases, the signal sequence is cleaved during or shortly after the polypeptide is transferred into the ER. Although the major requirement for a signal sequence is a stretch of at least 6–7 hydrophobic amino acids, there appear to be other, poorly defined properties that may distinguish different signal sequences [26,27]. For example, some signal sequences require auxiliary translocation components [28,29] or are sensitive to the drug cotransin [27,30]. In addition, signal sequences have other unexplained characteristics. For example, it has been noted that signal sequences in humans tend to be rich in leucine and poor in isoleucine [31], despite the fact that these two amino acids have similar hydrophobicities [32] and would thus be expected to function equally well to promote translocation. This leucine/isoleucine bias is not seen in prokaryotes. Another puzzling feature is that signal sequences are often conserved across species to a higher degree than expected [33]. These observations raise the possibility that not only the signal sequence per se, but also the encoding nucleotide sequence (signal sequence coding region or SSCR) may have a function.

We report here on the nuclear export of mRNAs coding for secretory proteins. We used microinjection of mRNAs and DNAs as well as transfection of DNA to demonstrate that the SSCR can promote the export of mRNAs that cannot use the conventional pathway because they lack an intron or a functional cap. By using large-scale sequence analysis, we found that vertebrate SSCRs have a low content of adenine, a feature that may in part be responsible for the preference of leucine versus isoleucine in signal sequences. Consistent with these observations, the incorporation of silent adenine mutations within the SSCR inhibits its nuclear export activity. Even the export of a natural RNA containing introns is facilitated by its SSCR. The discovery of an SSCR-mediated mRNA export pathway thus explains the previously noted amino acid bias in signal sequences and suggests a link between nuclear export and membrane targeting of mRNAs.

## Results

### Efficient Splicing and Translation of Microinjected t-ftz RNA

**Figure 1:**
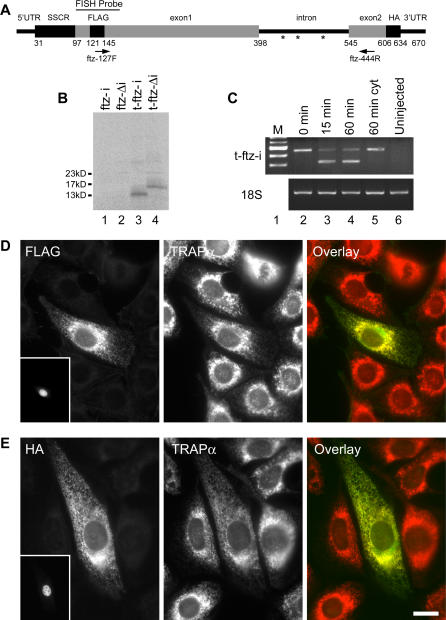
Microinjected t-ftz mRNA Is Spliced and Translated In Vivo (A) Scheme of the t-ftz transcript. The regions complementary to the oligonucleotides used for FISH (“FISH probe”) and for RT-PCR (“ftz-127F” and “ftz-444R”) are indicated. Asterisks represent in-frame stop codons within the intron. (B) The indicated transcripts were translated in vitro using reticulocyte lysate in the presence of ^35^S-methionine. Samples were separated by SDS-PAGE, and newly synthesized proteins were detected by autoradiography. (C) t-ftz-i was microinjected into either the nuclei (lanes 2–4) or cytoplasm (lane 5) of 20 NIH 3T3 cells, which were then incubated for the indicated times. RT-PCR was performed on the cell extracts using the intron flanking oligonucleotides indicated in (A). The lower band represents the spliced product, and the upper band represents the unspliced product. 18S rRNA was amplified and used as a loading control. Molecular weight markers (M) were loaded in lane 1. (D and E) NIH 3T3 cells were microinjected with t-ftz-i mRNA. FITC-conjugated 70-kDa dextran was co-injected to mark the injected compartment (insets). Cells were incubated for 4 h, fixed and immunostained for either FLAG (D) or HA (E) epitopes and for TRAPα. Overlays of the expressed epitope (green) and TRAPα (red) are shown in the last panel. Scale bar = 10 μm.

To study the nuclear export of mRNA coding for a secretory protein, we used a model mRNA that is derived from a fragment of the *fushi tarazu* (*ftz*) gene. The original construct contains an intron and was previously used to monitor mRNA splicing and nuclear export in *Xenopus* oocytes [1,34]. The construct was modified by adding a Kozak consensus sequence to allow efficient expression in mammalian cells. Sequences encoding FLAG and hemagglutinin (HA) epitopes were included at the 5′ and 3′ ends of the open reading frame (ORF), respectively, to monitor translation of the mRNA. Because the intron contains in-frame stop codons (Figure 1A; asterisks), the HA epitope will only be synthesized if the mRNA is spliced. To target the translation product to the ER, we attached an SSCR derived from the mouse major histocompatibility complex (MHC) class 2 molecule H2-K1. The final construct is called t-ftz-i (Figure 1A and Figure S1).

We first tested the translation of the t-ftz-i mRNA in vitro. When translated in reticulocyte lysate, a polypeptide of 13 kDa was generated, consistent with the size expected from the location of the in-frame stop codon in the intron (Figure 1B, lane 3). When the intron was deleted, the resulting mRNA (t-ftz-Δi) gave rise to a 17-kDa translation product (lane 4), again in agreement with the expected size of the ORF. With the original ftz constructs that lacked translation initiation signals, no translational products were detected (lanes 1, 2).

When t-ftz-i mRNA was microinjected into nuclei of NIH 3T3 fibroblasts, it was efficiently spliced within 15 min, as shown by reverse-transcriptase (RT)-PCR (Figure 1C; lanes 2–4). As expected, no detectable splicing was observed when t-ftz-i was microinjected into the cytoplasm (lane 5). Next, we tested the translation of t-ftz-i transcripts that were microinjected into the nuclei of NIH 3T3 fibroblasts. Nuclear injection was confirmed by co-injecting fluorescently labeled 70-kDa dextran, which is too large to passively cross the nuclear pores (Figure 1D and 1E; see insets). After 4 h, the FLAG and HA epitopes could be detected by immunofluorescence microscopy in over 90% of the injected cells (Figure 1D and 1E). In addition, both epitopes co-localized with the ER resident protein TRAPα (Figure 1D and 1E), indicating that the translation product was translocated into the ER. The protein is probably not secreted efficiently, because it contains only a fragment of ftz and therefore may not be properly folded. When t-ftz-i transcripts were injected into the cytoplasm, the expression of the FLAG but not of the HA epitope was observed (unpublished data), as expected from the presence of the unspliced intron. Both the FLAG and HA epitopes were expressed when t-ftz-Δi transcripts were injected into nuclei or cytoplasm (unpublished data).

### Nuclear mRNA Export Requires Splicing or the SSCR

**Figure 2:**
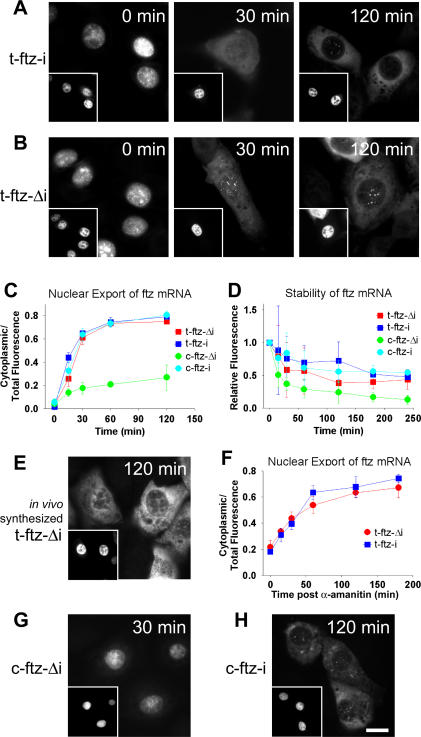
The SSCR Promotes Nuclear Export of mRNA (A and B) NIH 3T3 cells were microinjected with either t-ftz-i (A) or t-ftz-Δi (B) and FITC-conjugated 70-kDa dextran (insets). After the indicated time points, cells were fixed and probed for ftz mRNA by FISH. (C and D) Quantitation of the cytoplasmic/total FISH fluorescence (C) and total FISH fluorescence (D) from cells microinjected with the indicated mRNAs. Each data point represents the average of three experiments, each of which consisted of 15–30 cells. Error bars represent the standard deviation between the three experiments. Relative fluorescence in (D) was calculated by normalizing the total fluorescence at a given time point to the fluorescence at 0 min in each experiment. (E) A plasmid containing the t-ftz-Δi gene, and FITC-conjugated 70-kDa dextran (inset) were microinjected into NIH 3T3 cells. After 30 min, α-amanitin was added, and the cells were incubated for an additional 120 min. The cells were fixed and probed for ftz mRNA by FISH. (F) NIH 3T3 cells were microinjected with plasmids containing either the t-ftz-Δi or t-ftz-i genes. After 30 min, the cells were treated with α-amanatin and incubated for the indicated time points. The cells were then fixed and probed for ftz mRNA. Nuclear export was quantified as in (C). (G and H) NIH 3T3 cells were microinjected with either c-ftz-Δi (G) or c-ftz-i (H) and FITC-conjugated 70-kDa dextran (insets). After the indicated time points, the cells were fixed, and probed for ftz mRNA by FISH. Scale bar = 15 μm.

To monitor the nuclear export of t-ftz-i mRNA, transcripts were microinjected into nuclei of NIH 3T3 cells. The cells were fixed at various time points, and the localization of the injected RNA was probed by fluorescence in situ hybridization (FISH). We optimized the FISH procedure, omitting harsh acid and ethanol treatments, such that intracellular morphology was largely maintained. We estimate that 20,000 to 50,000 transcripts were injected per cell, which is a small number compared with the total number of transcripts in a typical mammalian cell (400,000 to 850,000 molecules) [35]. Again, fluorescent 70-kDa dextran was co-injected to identify nuclear-injected cells (Figure 2, insets). Immediately after injection, the transcripts were confined to the nucleus. Over time, however, the majority of t-ftz-i transcripts (∼80%) accumulated in the cytoplasm (Figure 2A). Quantitation showed that the half-time of mRNA export was ∼15 min (Figure 2C), similar to previous estimates [36,37]. About 50% of the mRNA molecules remained intact after 4 h, as shown by the total level of FISH signal (Figure 2D).

Surprisingly, when we injected t-ftz-Δi mRNA into nuclei, we also observed efficient export into the cytoplasm (Figure 2B; quantitation in Figure 2C), despite the fact that the absence of an intron should have prevented the recruitment of TREX components and thus nuclear export [2,4]. The kinetics of mRNA export was about the same for transcripts containing or lacking the intron (Figure 2C). In contrast to a previous report [38], a large fraction of the intron-less transcripts remained stable over the time of analysis (Figure 2D).

To exclude the possibility that the nuclear export of the intron-less transcript is caused by the introduction of exogenously synthesized mRNAs, we tested the export of mRNA after its synthesis in the nucleus. To this end, we microinjected plasmids containing the t-ftz-Δi or t-ftz-i genes into NIH 3T3 nuclei. After 30 min, the RNA Polymerase II inhibitor α-amanitin was added to inhibit further transcription, and then the distribution of mRNA over time was monitored using FISH. Immediately after α-amanitin addition, most transcripts were in the nucleus, but ∼20% were already found in the cytoplasm. Over time, the nuclear fraction was efficiently exported to the cytoplasm (Figure 2E and 2F) with a rate that was slightly lower than that of microinjected RNA. As before, we observed only minor differences between intron-containing and intron-lacking t-ftz transcripts (Figure 2F). Pretreatment of cells with α-amanitin 5 min prior to DNA microinjection completely inhibited mRNA synthesis, as assayed by FISH (unpublished data). From these results, we conclude that export of an intron-lacking mRNA can occur efficiently, regardless of whether or not export is coupled to transcription.

Our results are in apparent contradiction to previous observations showing that ftz constructs lacking introns are not exported from nuclei of *Xenopus* oocytes [1]. A major difference to the transcripts tested in *Xenopus* oocytes is the presence of an SSCR in t-ftz-Δi. We therefore tested whether the SSCR was required to promote nuclear export of this mRNA. Indeed, a transcript that lacked the SSCR, and thus encoded a cytoplasmic version of ftz (c-ftz-Δi), remained in the nucleus 30 min after injection (Figure 2G, quantitation in Figure 2C), a time during which most of the control t-ftz-Δi mRNA was exported. At later time points, much of the c-ftz-Δi mRNA was degraded (Figure 2D). Because c-ftz-Δi mRNA was stable when injected directly into the cytoplasm (unpublished data), it is unlikely that nuclear-injected c-ftz-Δi mRNA was exported and then rapidly degraded in the cytoplasm. The addition of an intron into c-ftz-Δi mRNA (resulting in c-ftz-i mRNA) restored nuclear export and stability of the mRNA (Figure 2H, quantitation in Figure 2C and 2D). A fraction of the exported c-ftz-i transcripts accumulated in stress granules (unpublished data). Together, these experiments suggest that either an SSCR or an intron can serve as a nuclear export signal.

**Figure 3:**
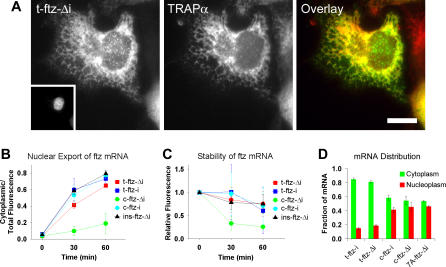
The SSCR Promotes Nuclear Export and ER Targeting of mRNA in COS-7 Cells (A) COS-7 cells were microinjected with t-ftz-Δi mRNA and FITC-conjugated 70-kDa dextran (inset) and then incubated for 90 min. The cells were fixed, probed for ftz mRNA by FISH, and immunostained for TRAPα. An overlay of the mRNA (green) and TRAPα (red) staining is shown in the right panel. Scale bar = 15 μm. (B and C) Quantitation of the cytoplasmic/total FISH fluorescence (C) and total FISH fluorescence (D) in COS-7 cells microinjected with the indicated mRNAs, as described in Figure 2C and 2D. (D) COS-7 cells were transfected with plasmids containing the indicated genes, incubated for 12–18 h, fixed, and probed for ftz mRNA. Cells were then imaged, and the cytoplasmic and nuclear fluorescence was quantified. Each bar represents the average of three experiments in which 20 cells were analyzed. Error bars represent the standard deviation between the three experiments.

To exclude the possibility that the SSCR-mediated export pathway is a peculiarity of NIH 3T3 cells, we microinjected mRNA precursors into the nuclei of COS-7 cells. As before, t-ftz-i mRNA containing both the splicing and the SSCR signals—as well as t-ftz-Δi and c-ftz-i mRNAs, which each contain only one of the two signals—were exported efficiently into the cytoplasm (Figure 3A and Figure S2; quantitation in Figure 3B). In contrast, c-ftz-Δi mRNA lacking both signals was not exported (Figure 3B and Figure S2). Again, the c-ftz-Δi mRNA was significantly less stable than the other mRNAs (Figure 3C). These data suggest that the SSCR-mediated nuclear export pathway may be present in many mammalian cell types.

In COS-7 cells, we were able to visualize the targeting of the mRNAs to the ER membrane. All mRNAs containing an SSCR gave a typical reticular staining in the cytoplasm and co-localized with TRAPα, a marker of the ER membrane (Figure 3A and Figure S2).

To test whether other SSCRs could promote mRNA export, we replaced the SSCR of the MHC class 2 molecule H2-K1 with that of human insulin in the t-ftz-Δi construct (ins-ftz-Δi, see Figure S3). When injected into COS-7 cell nuclei, ins-ftz-Δi mRNA was efficiently exported (Figure 3B and Figure S2). In fact, the export kinetics was faster than with t-ftz-Δi mRNA (Figure 3B). Again, the mRNA was targeted to the ER membrane (Figure S2). Taken together, these results indicate that the SSCR-mediated pathway may be quite general.

Finally, we performed transfection experiments in COS-7 cells with plasmids coding for t-ftz and c-ftz mRNAs containing or lacking an intron. The steady-state distribution of the mRNAs between the nucleus and cytoplasm was determined by FISH (Figure S4, quantitation in Figure 3D). The SSCR-containing mRNAs (t-ftz-i and t-ftz-Δi) were mostly found in the cytoplasm, indicating that they are efficiently exported from the nucleus. A significant fraction of mRNAs lacking an SSCR (c-ftz-i and c-ftz-Δi) were found in the nucleus, despite the fact that one contained an intron. In the cytoplasm, the SSCR-containing ftz constructs were targeted to the ER membrane, whereas c-ftz constructs partially co-localized with TIA-1 (Figure S4), a marker of stress granules [39]. These data confirm the presence of an SSCR-mediated mRNA export pathway and suggest that, at least under certain conditions, it may be more efficient than the splicing-mediated export pathway.

### Characterization of SSCR-Mediated mRNA Export

**Figure 4:**
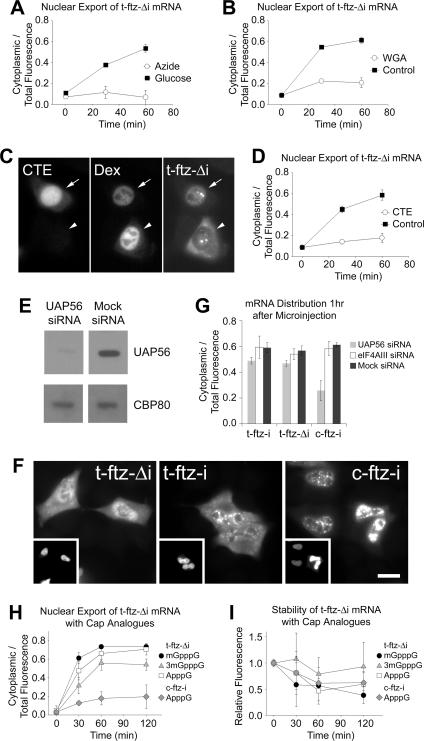
Characterization of SSCR-Mediated Export (A) NIH 3T3 cells were microinjected with t-ftz-Δi mRNA and then incubated in Dulbeco's modified PBS supplemented with either azide or glucose. After the indicated time points, the cells were fixed and probed for ftz mRNA. Quantification of nuclear export was performed as in Figure 2C. (B) NIH 3T3 cells were pre-injected with either wheat germ agglutinin (WGA) or with human IgG (control). After 30 min, the cells were microinjected with t-ftz-Δi mRNA and incubated for the indicated times. The cells were fixed and probed for ftz mRNA. Quantification of nuclear export was performed as in Figure 2C. (C) NIH 3T3 cells were pre-injected with CTE RNA and a marker, cascade blue–conjugated 10-kDa dextran. After 30 min, the cells were microinjected with t-ftz-Δi mRNA and FITC-conjugated 70-kDa dextran. After 1 h, the cells were fixed and probed for ftz mRNA. The arrow indicates a cell pre-injected CTE, which displayed a defect in the export of t-ftz-Δi mRNA. The arrowhead indicates a neighboring cell that was not pre-injected with CTE and showed normal export of t-ftz-Δi mRNA. (D) NIH 3T3 cells were either pre-injected with CTE and marker (CTE) or marker alone (control) and then 30 min later were microinjected with t-ftz-Δi mRNA. At the indicated time points, the cells were fixed and probed for ftz mRNA. Quantification of nuclear export was performed as in Figure 2C. (E) HeLa cells were treated with siRNA oligonucleotides directed against both UAP56 and URH49 or with control oligonucleotides (Mock). Cell lysates were collected and separated by SDS-PAGE. UAP56 and CBP80 were detected by Western blotting. (F and G) HeLa cells were treated for 24 h with siRNA against both UAP56 and URH49 (F and G) or for 48 h against eIF4AIII (G). The cells were then microinjected with either t-ftz-Δi, t-ftz-i, or c-ftz-i mRNA and FITC-conjugated 70-kDa dextran (insets). After 1 h, the cells were fixed, probed for ftz mRNA, imaged (F), and quantified for nuclear export (G). Each bar represents the average of three experiments, in which 20–30 cells were analyzed. Error bars represent the standard deviation between the three experiments. Scale bar = 15 μm. (H and I) Quantification of nuclear export and stability of microinjected t-ftz-Δi or c-ftz-i mRNA capped with either a natural cap (mGpppG), trimethylated cap (3mGpppG), or adenine cap (ApppG). At the indicated time points, the microinjected cells were fixed and probed for ftz mRNA. Quantitation of nuclear export (H) and of the total fluorescence (I) was performed as in Figure 2C and 2D, respectively.

Next we characterized SSCR-mediated mRNA export, using t-ftz-Δi mRNA that contains the SSCR signal but lacks an intron. When this mRNA was injected into nuclei of NIH 3T3 cells that had been depleted of ATP by treatment with azide [40], no export into the cytoplasm was observed (Figure 4A). This treatment did not compromise the viability of the cells as judged by phase microscopy or immunostaining of the microtubule network (unpublished data). Blocking the nuclear pores by pre-injecting wheat germ agglutinin (WGA) [41,42] also inhibited the nuclear export of microinjected t-ftz-Δi mRNA (Figure 4B). Thus the export of t-ftz-Δi mRNA requires energy and functional nuclear pores.

As expected, nuclear mRNA export was unidirectional. When transcripts were injected into one of the nuclei of a binucleated cell, 80% of the mRNA was transported into the cytoplasm, but no fluorescence was detected in the uninjected nucleus (Figure S5), indicating that there is no re-uptake of exported mRNA.

Next we attempted to define the components required for t-ftz-Δi mRNA export. To test whether the export factor TAP is required, we took advantage of the viral constitutive transport element (CTE), which binds to and sequesters TAP [43]. Nuclear export of t-ftz-Δi mRNA was inhibited in cells pre-injected with CTE RNA (Figure 4C, arrow indicates a cell pre-injected with CTE, quantitation is shown in Figure 4D). In contrast, cells that were not pre-injected with CTE (Figure 4C, arrowhead), or cells pre-injected with control buffer (Figure 4D), exported t-ftz-Δi mRNA. Previously, it was demonstrated that short mRNAs use the TAP-independent snRNA export pathway [44], but our results demonstrate that the t-ftz-Δi transcripts exclusively follow the TAP-dependent pathway used by most mRNAs.

We then tested the involvement of the TREX complex. To deplete the TREX complex, we incubated HeLa cells with small interfering RNAs (siRNAs) against the TREX component UAP56 and its ortholog URH49, a treatment that has been shown to inhibit the majority of mRNA export in mammalian cells [45]. After one day of RNA interference (RNAi) treatment, UAP56 levels were effectively reduced (Figure 4E). Although siRNA-treated cells were compromised for the export of c-ftz-i transcript, these cells exported both t-ftz-Δi and t-ftz-i mRNA (Figure 4F and 4G), suggesting that the SSCR-mediated export pathway is less sensitive to TREX complex depletion than the splicing-mediated pathway. In some cells depleted of UAP56/URH49, t-ftz-Δi and t-ftz-i transcripts became enriched in the nuclear rim, perhaps due to a pleiotropic effect that inhibits the movement of mRNAs from the nuclear pores into the cytoplasm. Cells treated with siRNA for two days showed even more nuclear rim localization of t-ftz-Δi and t-ftz-i transcripts, and their export was inhibited. Although c-ftz-i mRNA was also not exported, it only accumulated in the nucleoplasm (unpublished data), supporting the idea that different export pathways are used.

As expected, the export of the intron-lacking RNA t-ftz-Δi was not reduced when a component of the exon junction complex (EJC), the RNA helicase eIF4AIII, was depleted by RNAi (Figure 4G and Figure S6). However, even the export of the intron-containing mRNAs t-ftz-i and c-ftz-i remained unaffected, in agreement with previous results that indicated that the EJC is not a main factor in recruiting TREX [3,46].

Finally, we tested whether the SSCR-mediated export requires a 5′ end cap, as in splicing-dependent export [3]. Because uncapped transcripts were rapidly degraded after microinjection into NIH 3T3 nuclei (unpublished data), we used mRNAs that were capped with the cap analogs ApppG or trimethyl-2,3,7-GpppG (3mGpppG). These modified caps do not associate with the cap binding complex CPB80/20 [47], which is essential for the subsequent recruitment of the TREX complex [3] and for efficient splicing [47]. Capping of t-ftz-Δi mRNAs with the cap analogs did not inhibit nuclear export (Figure 4H). In addition, the mRNAs were as stable as transcripts containing the natural cap, methyl-7-GpppG (mGpppG) (Figure 4I). In contrast, the c-ftz-i transcripts that use the splicing-dependent pathway were not exported when capped with ApppG, as expected (Figure 4H). Thus, we conclude that unlike the splicing-mediated mRNA export, the SSCR-mediated pathway does not require a natural cap.

### The SSCR Nuclear Export Signal Is an RNA Element

**Figure 5:**
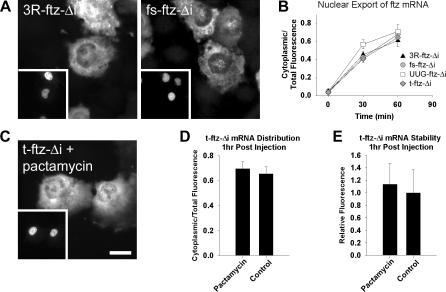
The SSRC Is an RNA Element That Promotes Nuclear Export (A and B) COS-7 cells were microinjected with either 3R-ftz-Δi, fs-ftz-Δi, UUG-ftz-Δi, or t-ftz-Δi mRNA and FITC-conjugated 70-kDa dextran (insets) and then incubated for 60 min (A) or the indicated time points (B). Cells were fixed and probed for ftz mRNA. Quantitation of the nuclear export (B) was performed as in Figure 2C. (C–E) COS-7 cells were pretreated with either pactamycin or DMSO (control) for 20 min and then microinjected with t-ftz-Δi mRNA. After 1 h the cells were fixed and probed for ftz mRNA (C). Quantitation of the nuclear export (D) and stability (E) were carried out as in Figure 2C and 2D, except that the relative fluorescence (E) was calculated by normalizing the fluorescence to that of the control treated cells. Scale bar = 15 μm.

In principle, the SSCR nuclear export signal could either be an RNA element or its translated amino acid sequence, the signal sequence. To distinguish between these possibilities, we altered five nucleotides within the SSCR such that three encoded hydrophobic residues within the core of the signal sequence were changed to arginines (3R-ftz, see Figure S3). The mutated signal sequence should no longer target the translation product to the ER membrane. Upon injection into the nuclei of COS-7 cells, 3R-ftz-Δi mRNA was exported with a kinetics similar to that of t-ftz-Δi (Figure 5A; quantitation in Figure 5B). As expected, the mRNA was no longer targeted to the ER, and instead was distributed diffusely in the cytoplasm (Figure 5A). Its translation product was targeted to mitochondria (unpublished data), possibly because the signal sequence was converted into an amphipatic helix typical of mitochondrial targeting sequences [48].

To further ensure that the translation product of the SSCR did not determine mRNA export from the nucleus, we altered the ORF of the SSCR. We added a nucleotide at the beginning of the sequence and deleted a nucleotide at the end, such that the coding region following the SSCR remained unchanged. The altered SSCR codes for a less hydrophobic sequence. The frame-shifted ftz transcript (fs-ftz-Δi, Figure S3) was again efficiently exported into the cytoplasm (Figure 5A and 5B). As expected, it was not targeted to the ER (Figure 5A), and its translation product remained in the cytoplasm (unpublished data). Transcripts that lacked a translation start codon at the beginning of the SSCR (UUG-ftz-Δi, see Figure S3) were also efficiently exported (Figure 5B and Figure S2).

To further confirm that translation of the t-ftz-Δi transcript was not required for its export, cells were pretreated with pactamycin, an inhibitor of translation initiation [49], before being microinjected. Although translation was effectively inhibited after 15 min of drug treatment, as assayed by ^35^S-methionine incorporation (unpublished data), an even longer pretreatment with pactamycin (20 min) did not inhibit t-ftz-Δi mRNA export or affect t-ftz-Δi stability (Figure 5C–5E). Interestingly, after 1 h of pactamycin pretreatment, mRNA export was inhibited and nuclear rim staining was seen, as in cells depleted of the TREX components UAP56 and URH49, which suggests that in both conditions, the synthesis of a factor required for the movement of the mRNA from the nuclear pores into the cytoplasm is impaired.

From these experiments, we conclude that the nuclear export signal of the SSCR does not require translation and is thus likely an RNA element.

### SSCRs in Vertebrates Are Deficient in Adenines

**Figure 6:**
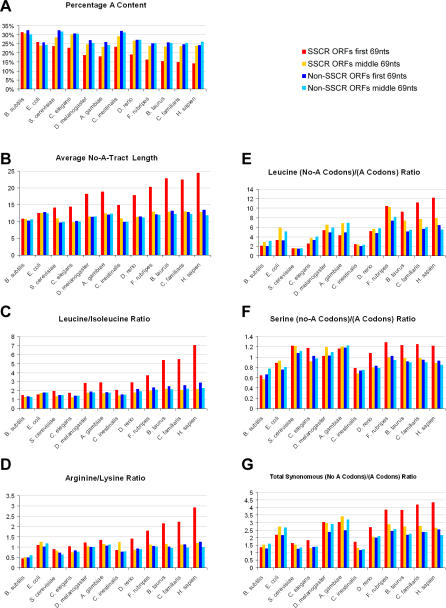
Large-Scale Genomic Analysis of the SSCR All analysis was performed on four data sets from each genome; the first 69 nucleotides (nts) after the start codon of SSCR-containing ORFs (red bars), the middle 69 nts of SSCR-containing ORFs (yellow bars), the first 69 nts from ORFs lacking a SSCR (dark blue bars), and the middle 69 nts from this set of ORFs (light blue). Each bar represents the average across the entire set of sequences within a given genome. (A and B) For each sequence the overall percentage of adenine (A) and the longest tract lacking an adenine (no-A-tract) (B) was calculated for each ORF and then averaged over each set. (C and D) The average ratio of encoded leucines to isoleucines (C), and encoded arginines to lysines (D) was calculated. (E and F) The average ratio of [CUU, CUC, CUG, UUG]/[CUA, UUA] codons for leucine (E), and [UCU, UCC, UCG]/[UCA, AGU, AGC] codons for serine (F) was calculated. (G) The codons for leucine, valine, serine, proline, alanine, argenine, and glycine were compiled, and the average ratio between the adenine-lacking codons to adenine-containing codons was calculated.

To identify features in SSCRs that might function as nuclear export signals, we performed a large-scale sequence analysis of various genomes. We confined our analysis to the first 69 base pairs (bp) following the initiator methionine codon, a length that covers most SSCRs. We used the annotation in Ensembl and PSORTdb to classify genes into SSCR-containing and SSCR-lacking ORFs in genomes ranging from bacteria to humans. As an additional control, we analyzed 69 bp in the central region of each ORF, which should reflect the overall base composition of the coding region. This analysis showed that the SSCRs in all eukaryotes have a marked deficiency of adenines, in contrast to those in bacteria (Figure 6A). Consistent with this observation, eukaryotic SSCRs have long nucleotide stretches devoid of adenines (no-A tracts) (Figure 6B). The no-A tracks were longer in vertebrates than in invertebrates.

Several factors account for the adenine deficiency in SSCRs. First, hydrophobic amino acids, which are enriched in the signal sequence, have codons that contain few adenines. Another factor is bias among amino acids with similar biochemical properties, such that amino acids encoded by codons with fewer adenines are used. This is illustrated by leucine, which has few adenine-containing codons, and isoleucine, which has at least one adenine in each of its codons. As previously noted, human signal sequences have significantly more leucine residues than equally hydrophobic isoleucine residues [31]. We confirmed this leucine-versus-isoleucine bias for SSCRs in all vertebrates analyzed (Figure 6C). A similar analysis was performed on positively charged amino acids that are frequently found in the amino acid sequence preceding the hydrophobic core of a signal sequence. Arginine is encoded by codons containing relatively few adenines, whereas lysine is encoded by AAA and AAG, and arginines were significantly more frequent than lysines in vertebrate, but not invertebrate, SSCRs (Figure 6D). In total, about 15% to 25% of the adenine deficiency in SSCRs in vertebrates is caused by selection between similar amino acids for those encoded by codons with lower adenine content (Figure S7). The third factor that contributes to the adenine deficiency is a bias toward codons lacking adenine for amino acids that are encoded by multiple codons (e.g., CUC versus CUA). This is illustrated by the usage of codons for leucine and serine. In vertebrates, the adenine-lacking codons for both amino acids are more frequently used than the ones containing adenines (Figure 6E and 6F). The bias against adenine-containing codons in vertebrate SSCRs could also be seen when the analysis was extended to all amino acids that are encoded by both adenine-lacking and -containing codons (Figure 6G). In total, bias between synonymous codons accounts for an additional 15% to 25% of the adenine deficiency in vertebrates (Figure S7). In humans, the bias between similar amino acids and between synonymous codons together account for almost half of the total adenine deficiency (Figure S7). Our analysis suggests that over the course of evolution, there was a strong selection against adenines that was likely caused by some requirement of the nucleotide sequence, rather than of the encoded amino acid sequence.

It should be noted that the two SSCRs that were tested experimentally, completely follow the rules established in the large-scale sequence analysis. The longest no-A track in the SSCRs derived from H2-K1 and insulin were 35 and 40 nucleotides, respectively. Both SSCRs contain leucines and arginines, but no isoleucines or lysines, and both have a high bias against adenine-containing codons.

### Adenine-Containing SSCRs Do Not Promote Efficient mRNA Export

**Figure 7:**
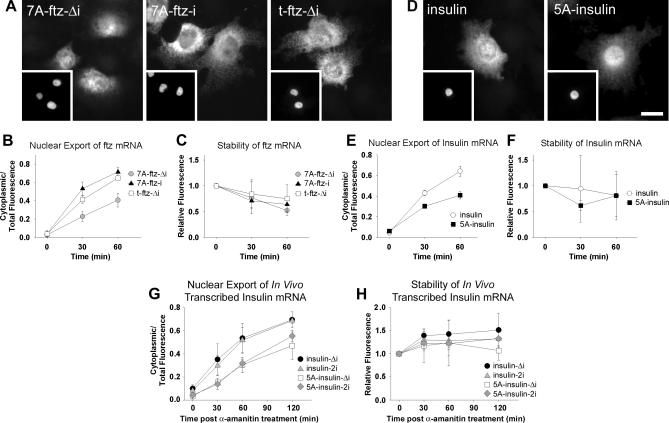
Silent Adenine Mutations Inhibit SSCR-Dependent Nuclear Export of mRNA (A–C) COS-7 cells were microinjected with t-ftz transcripts containing seven silent adenine mutations within the SSCR. FITC-conjugated 70-kDa dextran was co-injected as a marker (insets). Cells were incubated for 1 h (A) or various time points (B and C) to allow for export, then fixed and probed for ftz mRNA (A). Quantification of nuclear export (B) and stability (C) were performed as in Figure 2C and 2D. (D–F) As in (A–C), except that COS-7 cells were microinjected with either wild-type insulin transcripts or an insulin transcript containing five silent adenine mutations within the SSCR (5A-insulin). FITC-conjugated 70-kDa dextran was co-injected (insets). Cells were incubated for 1 h (D) or various time points (E and F) to allow for export, then fixed and probed for ftz mRNA (D). Quantification of nuclear export (E) and stability (F) as in Figure 2C and 2D. Scale bar = 15 μm. (G and H) COS-7 cells were microinjected with plasmids containing the indicated genes. After 30 min, the cells were treated with α-amanatin and incubated for the indicated time points. The cells were then fixed and probed for ftz mRNA. Quantification of nuclear export (G) and stability (H) were performed as in Figure 2C and 2D, except that each data point represents the average of four experiments, each of which consisted of 20–40 cells. Error bars represent the standard deviation between the four experiments.

To test whether the bias against adenines within SSCRs is important for promoting mRNA export, we mutated seven nucleotides of the SSCR that were derived from the MHC class 2 molecule H2-K1 to adenines, without altering the encoded amino acid sequence (7A-ftz-Δi, see Figure S3). Upon injection into COS-7 cell nuclei, the export of 7A-ftz-Δi mRNA into the cytoplasm was significantly less efficient than that mediated by the original SSCR (Figure 7A and 7B). The nuclear export of an intron-containing version (7A-ftz-i) was not inhibited by the mutations (Figure 7A and 7B), which is consistent with the expectation that this transcript can use the splicing-dependent pathway. The exported 7A-ftz-Δi and 7A-ftz-i mRNAs were targeted to the ER (Figure 7A) and resulted in the expression of ER-bound t-ftz protein (unpublished data), indicating that translation of the mRNAs was largely unaffected by the silent mutations. The mutations also did not grossly affect the stability of the mRNAs (Figure 7C). The nuclear export of 7A-ftz-Δi mRNA was also significantly less efficient than that of t-ftz-Δi mRNA when tested in transfection experiments with plasmids coding for these RNAs (Figure 3D and Figure S4). In fact, the export was as inefficient as with RNA lacking an SSCR altogether, indicating that the adenine mutations severely affected the SSCR-export signal. In addition, a portion of the cytoplasmic fraction of the 7A-ftz-Δi mRNAs was localized to stress granules, as determined by co-staining with the stress granule marker TIA-1 (Figure S4).

An inhibitory effect of adenines on the nuclear export function of an SSCR could also be demonstrated for human insulin mRNA. We first used intronless transcripts, generated by in vitro transcription of human insulin cDNA. Upon injection into COS-7 cell nuclei, insulin mRNA with a wild-type SSCR was efficiently exported into the cytoplasm, whereas mutant mRNA, in which five silent adenine mutations were introduced into its SSCR, was significantly delayed (Figure 7D and 7E). Again, the mutations did not significantly affect the stability of the mRNAs (Figure 7F).

To address whether the SSCR contributed to the export of a physiologically transcribed and spliced mRNA, we microinjected plasmids that contained the insulin gene with its two introns under the control of the CMV promoter (insulin-2i). After 30 min, transcription was blocked with α-amanitin, and nuclear export of the newly synthesized transcripts was followed over the course of 2 h. The rate of mRNA export was significantly decreased when silent adenine mutations were incorporated into the SSCR of intron-containing or -lacking mRNAs (5A-insulin-2i and 5A-insulin-Δi) (Figure 7G). On the other hand, deletion of the introns (insulin-Δi) had no effect. All the tested transcripts were stable over the tested time course (Figure 7H). From these experiments we conclude that a functional SSCR can enhance the export of mRNAs, regardless of whether they contain or lack introns. These results provide evidence that the SSCR-mediated pathway operates within the context of a natural gene.

## Discussion

Here we describe the surprising discovery of a nuclear export pathway in mammalian cells that appears to be specific for mRNAs coding for secretory proteins. We found that mRNAs lacking an intron or functional cap, which cannot use the canonical, splicing-dependent pathway, can efficiently be exported into the cytoplasm by means of a signal in the SSCR. The signal is an RNA element that is deficient in adenines. Thus, the SSCR not only codes for the hydrophobic amino acid sequence that targets the translation product to the ER membrane, but also functions at the nucleotide level to promote the export of the mRNA from the nucleus. The SSCR even enhances the export of natural transcripts containing introns. Like the splicing-dependent pathway, the SSCR-mediated export pathway requires the export factor TAP, but it is less dependent, or perhaps even independent, of the TREX complex. Our sequence analysis suggests that the SSCR-mediated pathway may exist in all vertebrates.

Why would mRNAs coding for secretory proteins use a separate nuclear export pathway? One possibility is that nuclear export of these mRNAs might be coupled with some downstream event in the cytoplasm. For example, it is possible that the mRNAs emerging from the nuclear pores are initially associated with proteins that keep them translationally silenced and promote their distribution in the cytoplasm. These factors could be distinct from those used by mRNAs coding for cytoplasmic proteins. For example, factors associated with SSCR containing transcripts could have affinity for ER membrane proteins or molecular motors. The recent observation of an association of the TAP homolog, NXF2, with kinesin lends support to the idea that the export and cytoplasmic distribution of mRNAs may be coupled [50]. Our own data show that in transfection experiments in which the steady-state distribution of mRNAs was investigated, transcripts with defective or lacking SSCRs were not only inefficiently exported but also often accumulated in cytoplasmic stress granules, indicating that their normal cytoplasmic distribution was disrupted (Figure S4). How nuclear mRNA export would be coupled with downstream events remains to be elucidated. It is also possible that the export of mRNAs coding for secretory proteins is regulated differently from other mRNAs in certain situations. However, we have not found any changes of SSCR-mediated mRNA export upon accumulation of misfolded proteins in the ER (unpublished data). In yeast, a block in secretion results in the relocalization of certain nuclear pore components to the cytoplasm and leads to the inhibition of protein transport between the nucleus and the cytoplasm [51,52]. Conceivably, this mechanism may also provide a feedback signal between the demand of secretory protein synthesis in the cytoplasm and the nuclear export of the corresponding SSCR-containing mRNAs.

The mechanism by which SSCRs are recognized and trigger nuclear mRNA export also remains to be investigated. A clear nucleotide motif that is common among all SSCRs is not obvious. One possibility is that a negative export regulator would bind to all adenine-containing, non-SSCR sequences. However, we favor models in which either the paucity of adenines per se is recognized by an export-mediating protein with a similar RNA-binding specificity as the Muscleblind family of proteins [53], or that the adenine-lacking segment of an SSCR folds into a conformation that would recruit an export factor. Regardless of the precise signal, its position at the 5′ end of the mRNA would allow this part of the mRNA to emerge into the cytoplasm first, in the same direction of mRNA export used in the splicing-dependent pathway [3,54]. Although many SSCR-containing mRNAs also contain introns, the 5′ end localization of the SSCR signal might allow it to recruit factors to the newly synthesized transcript before the introns are synthesized and thereby overrule the splicing-dependent signals.

Our data indicate that the SSCR recruits TAP, likely without involvement of TREX. One possibility is that the serine/arginine-rich (SR) proteins serve as adaptors for TAP binding. SR proteins associate with mRNAs during transcription and/or splicing. They are required for splicing [55], can associate with TAP [56,57], and are involved in the export of intron-lacking histone H2A mRNA [58]. We have not been able to prevent SSCR-dependent mRNA export by pre-injection of an SR antibody that inhibits splicing [55] (unpublished results), but further work is required to test the possible role of the SR proteins in SSCR-mediated export.

Our analysis shows that the SSCRs have a nucleotide bias that cannot be explained solely by the encoded amino acid sequence. Although these results indicate that the nucleotide sequence itself is important, there may be additional variability of signal sequences at the level of amino acids, as suggested by experiments in which different signal sequences responded differently to the accumulation of unfolded proteins in the ER [27]. One would assume that the SSCR-mediated mRNA export pathway operates in all eukaryotic cells, but our large-scale sequence analysis showed a clear bias against adenine-containing codons only in vertebrate SSCRs. A slight bias against adenines was seen in lower eukaryotes, but it is possible that SSCRs have additional properties that are found in all eukaryotes, for example, a common folded structure. The same argument might explain the absence of any obvious adenine bias at the 5′ end of genes coding for membrane proteins that lack a signal sequence (unpublished data), even though the corresponding mRNAs also need to be targeted to the ER and would likely take the same pathway as those coding for secretory proteins. Obviously, the surprising discovery of an SSCR-dependent export pathway for mRNAs raises a large number of interesting questions that need to be addressed in the future.

## Materials and Methods

### RT-PCR and plasmid constructs.

For RT-PCR, total RNA was extracted from injected cells and analyzed using M-MLV reverse transcriptase (Invitrogen) and HiFi Taq polymerase (Invitrogen) according to the manufacturer's protocol. PCR reactions were carried out using gene-specific primer pairs, Ftz-127F and Ftz-444R (see Figure 1A), and for controls, we used mouse 18S rRNA-448F and mouse 18S rRNA-926R.

The ftz constructs were modified to generate t-ftz-i (Figure 1A and Figure S1), t-ftz-Δi, and their derivatives (Figure S3). For in vivo mammalian expression experiments, t-ftz constructs were digested with HindIII and XhoI, and then ligated into the pcDNA3 expression vector. Human insulin cDNA was amplified from a cDNA library and the insulin gene was amplified from HeLa genomic DNA using gene-specific primer pairs digested with HindIII and XhoI, and then ligated into the pcDNA3 expression vector. Insulin was modified with PCR primers to generate 5A-insulin (see Figure S3).

###  RNA synthesis, purification, and in vitro translation.

In vitro transcription was carried out using the T7 mMESSAGE mMACHINE transcription kit containing excess cap (Ambion). To synthesize mRNAs with cap analogues, the reaction was carried out with 35 mM ApppG or 3mGpppG (New England Biolabs); 10 mM ATP, UTP, and CTP; and 1 mM GTP. Transcripts were poly-adenylated using Poly(A) tailing kit (Ambion), generating poly(A) tails of 200–300 nucleotides. mRNA purification was carried out using MEGAclear kit (Ambion). mRNA was then precipitated with 150 mM potassium acetate (pH 5.5) and 2.5 volumes 100% ethanol. mRNA was resuspended in injection buffer (100 mM KCl, 10 mM HEPES, pH 7.4). mRNAs were translated in a TnT reticulocyte lysate system in the presence of ^35^S-methionine (Promega).

### Cell culture, siRNA and DNA transfection, and microinjection.

NIH 3T3 fibroblasts were maintained in DMEM supplemented with 10% calf serum. HeLa and COS-7 cells were maintained in DMEM supplemented with 10% fetal bovine serum. Cells were plated overnight on 35-mm-diameter dishes with glass coverslip bottoms (MatTek Corp.). For RNAi experiments, HeLa cells were transfected with siRNA directed against human UAP56 and URH49 [45] or eIF4AIII [59]. 24 and 48 h post transfection, cells were either plated on 35-mm dishes (for microinjections) or collected to assess protein levels by SDS-PAGE and Western blot with rabbit anti-UAP56 serum [4], rabbit anti-eIF4AIII [59], or rabbit anti-CBP80 serum [3]. For DNA transfections, cells were transfected with DNA and lipofectamine (Invitrogen) using the manufacture's protocol.

Microinjections were performed as previously described [60]. mRNA was microinjected at 200 μg/ml along with fluorescein isothiocyanate (FITC)–conjugated 70-kDa dextran (1 mg/ml; Invitrogen). Insulin mRNA was heated to 70 °C for 10 min prior to injections. DNA was injected at 50 μg/ml along with FITC-conjugated 70-kDa dextran, and translation was inhibited with 50 μg/ml α-amanitin (Sigma). For export inhibition experiments, cells were first microinjected with WGA (3 mg/ml; Sigma) or CTE RNA (200 μg/ml) along with cascade-blue–conjugated 10-kDa dextran (Invitrogen), and then incubated for 30 min at 37 °C prior to mRNA microinjection. For azide treatments, microinjected cells were washed three times with Dulbecco's modified PBS (D-PBS; 10 mM phosphate, pH 7.4, 140 mM NaCl, 3 mM KCl, 0.8 mM CaCl_2_, 0.7 mM MgCl_2_) and incubated in D-PBS supplemented with 10 mM azide (Sigma) or 10 mM D-glucose (Sigma). For pactamycin treatments, cells were incubated in DMEM (10% fetal bovine serum) with 200 nM pactamycin 20 min before microinjections.

### Immunostaining and FISH.

Microinjected cells were washed with D-PBS, fixed in 4% paraformaldehyde (Electron Microscope Sciences) in D-PBS, and permeabilized with 0.1% Triton X100 (Peirce) in PBS. For immunostaining, fixed samples were first incubated with primary antibodies (rabbit polyclonal against TRAPα [61], goat polyclonal antibody against TIA-1 [Santa Cruz Biotechnology], 12CA5 monoclonal antibody against HA [Roche Applied Sciences], and M2 monoclonal antibody against FLAG [Sigma]) diluted 1:200 in immunostain solution (PBS, 0.1% Triton X100, 2 mg/ml RNAse free BSA; Ambion) for 30 min, washed three times with PBS, and incubated with various Alexa-conjugated secondary antibodies (Invitrogen), diluted 1:200 in immunostain solution. For FISH, fixed cells were washed with 50% formamide in 1X SSC (150 mM NaCl, 15 mM NaCitrate, pH 7.10) and then incubated overnight at 37 °C in 200-ml hybridization buffer (50% formamide, 100 mg/ml dextran sulphate, 0.02 mg/ml RNAse free BSA, 1 mg/ml Escherichia coli tRNA, 5 mM VRC, 1X SSC) containing 30–50 ng oligonucleotide probe (GTCGAGCCTGCCTTTGTCATCGTCGTCCTTGTAGTCACAACAGCCGGGACAACACCCCAT for ftz, GGTCCTCTGCCTCCCGGCGGGTCTTGGGTGTGTAGAAGAAGCCTCGTTCCCCGCACACTA for insulin) labeled at their 5′ end with Alexa-546 (Integrated DNA Technologies). Cells were washed in five times with 50% formamide in 1X SSC and images were captured using an EM-CCD Camera, Model C9100–12 (Hamamatsu) on an inverted microscope (200M, Carl Zeiss) using Metamorph software (Molecular Devices Corporation). Unaltered 14-bit images were quantified in Metamorph and analyzed in Excel (Microsoft). For each image the area (*A*) and the average intensity (*I*) of each injected nuclei (*n*) and cell body (*b*) were recorded. For the background intensity, the average intensity of an un-injected cell (*u*) was used. The cytoplasmic fluorescence was equal to (*Ab*)(*Ib* – *Iu*) – (*An*)(*In* – *Iu*). The ratio of cytoplasmic/total fluorescence equals [(*Ab*)(*Ib* – *Iu*) – (*An*)(*In* – *Iu*)]/[(*Ab*)(*Ib* – *Iu*)]. For figure production, the contrast and brightness of the aquired 14-bit micrographs were adjusted to optimize the ability to view the fluorescence. The resulting images were converted to 8-bit files using Metamorph.

### Analysis of SSCR sequence.

Nucleotide sequences were downloaded from Ensembl Biomart (http://www.biomart.org/index.html) and the National Center for Biotechnology Information (NCBI) (*E. coli.* and Bacillus subtilis). Signal sequence containing proteins were determined by annontation in PSORTdb (E. coli and B. subtilis) and Ensembl Biomart. A Perl script (Protocol S1) was written to count the nucleotide content, amino acid content, and codon content, of the first 69 nucleotides, middle 69 nucleotides (offset toward the start to maintain frame as needed), and last 69 nucleotides. The script also determined the longest no-adenine and one-adenine tracks completely contained in the first 69 nucleotides. Tabulated results were examined in Excel to determine nucleotide content, codon bias, and amino acid bias. Percent of adenine bias explained by codon bias (e.g., CUC versus CUA) and similar amino acid bias (e.g., isoleucine versus leucine) was calculated by comparing the number of adenines in non–signal sequence containing proteins to the number of adenines found in signal sequence containing proteins normalized for the number of proteins.

## Supporting Information

Figure S1 Translocated ftz-i Sequence Note that the sequence of the intron is underlined.
(20 KB DOC)

Figure S2 The SSCR Promotes Nuclear Export and ER Targeting of mRNA in COS-7 Cells COS-7 cells were microinjected with the indicated mRNA and FITC-conjugated 70-kDa dextran (inserts) and then incubated for 60 min. Cells were fixed and probed for ftz mRNA.
(6.8 MB EPS)

Figure S3 Nucleotide and Amino Acid Sequences of SSCR Mutant Constructs Note that the longest no-A tracts in the H2-K1 and insulin SSCRs are underlined and that point mutations and resulting amino acid changes are in bold. For fs-ftz, the nucleotide addition is in bold, whereas the position of the nucleotide deletion is represented by a dash.
(24 KB DOC)

Figure S4 mRNAs Containing SSCRs Are Efficiently Exported and Do Not Accumulate in Cytoplasmic Stress Granules COS-7 cells were transfected with plasmids containing the indicated genes, incubated for 12–18 h, fixed, and probed for ftz mRNA by FISH (top row, green in overlay) and TIA-1 protein by immunostaining (middle row, red in overlay). Note that a portion of the cytoplasmic c-ftz-Δi, c-ftz-i, and 7A-ftz-Δi accumulated in cytoplasmic foci that were enriched in TIA-1. Scale Bar = 15 μm.
(18.9 MB EPS)

Figure S5 mRNA Export Is Unidirectional t-ftz-Δi transcript and FITC-conjugated 70-kDa dextran (inset) were microinjected into a single nucleus of a binucleate NIH 3T3 cell. After 3 h, the cell was fixed, probed for ftz mRNA, and imaged. Note that although t-ftz-Δi mRNA was exported from the injected nucleus (labeled “Inj”) into the cytoplasm, it did not get imported into the uninjected nucleus (labeled “U”).
Scale Bar = 15 μm.
(2.5 MB EPS)

Figure S6 eIF4AIII siRNA Treatment HeLa cells were treated with siRNA oligonucleotides directed against eIF4AIII or with control oligonucleotides (Mock) for 48 h. Cell lysates were collected and separated by SDS-PAGE. eIF4AIII and UAP56 were detected by Western blotting.
(1.3 MB EPS)

Figure S7 The Percent Adenine Deficiency in the SSCR Ascribed to Codon Bias and Similar Amino Acid Bias To calculate the decrease in adenine percentage in the first 69 nucleotides of SSCR-containing ORFs due to codon bias, the difference between the expected number of adenines in the SSCR, and the actual number of adenines caused by codon bias in the SSCR (see Figure 6G) was divided by the total decrease in adenines (see Figure 6A). To calculate the decrease in adenine due to similar amino acid bias, the drop in adenine levels caused by various amino acid substitutions in the SSCR (leucine for isoleucine [see Figure 6C], arginine for lysine [see Figure 6D], serine for threonine, aspartate for glutamate, glutamine for asparagine, and cystein for methionine [excluding the start codon]) was divided by the total decrease in adenines. The remaining drop in adenine percentage was ascribed to the prevalence of hydrophobic amino acids in the SSCR.
(51.7 MB EPS)

Protocol S1 SSCR Analysis Program Perl script to determine the nucleotide, amino acid, and codon content of the first 69 and middle 69 nucleotides (offset toward the start to maintain frame as needed). The script also determined the longest no-adenine and one-adenine tracks completely contained in the each 69-nucleotide segment.
(11 KB TXT)

### Accession Numbers

The GenBank (http://www.ncbi.nlm.nih.gov/Genbank) accession numbers for genes investigated in the paper are as follows: H2-K1 (GI: 14972), TAP (GI: 10482), UAP56 (GI: 7919), URH49 (GI: 10212), and eIF4AIII (GI: 9775). The GenBank accession number for human insulin cDNA is GI: 3630.

## Abbreviations

CTE: constitutive transport element
D-PBS: Dulbecco's modified phosphate-buffered saline
EJC: exon junction complex
ER: endoplasmic reticulum
FISH: fluorescence in situ hybridization
*ftz*: *fushi tarazu*
HA: hemagglutinin
MHC: major histocompatibility complex
ORF: open reading frame
SSCR: signal sequence coding region
TREX: transcription export
