# Supplementary material for: The Signal Sequence Coding Region Promotes Nuclear Export of mRNA
Source: PLoS Biol. 2007 Dec 4;5(12):e322. doi: 10.1371/journal.pbio.0050322 (PMC2100149; doi:10.1371/journal.pbio.0050322)
Supplement: Supplementary file 1 [file pbio.0050322.sg007.pdf]

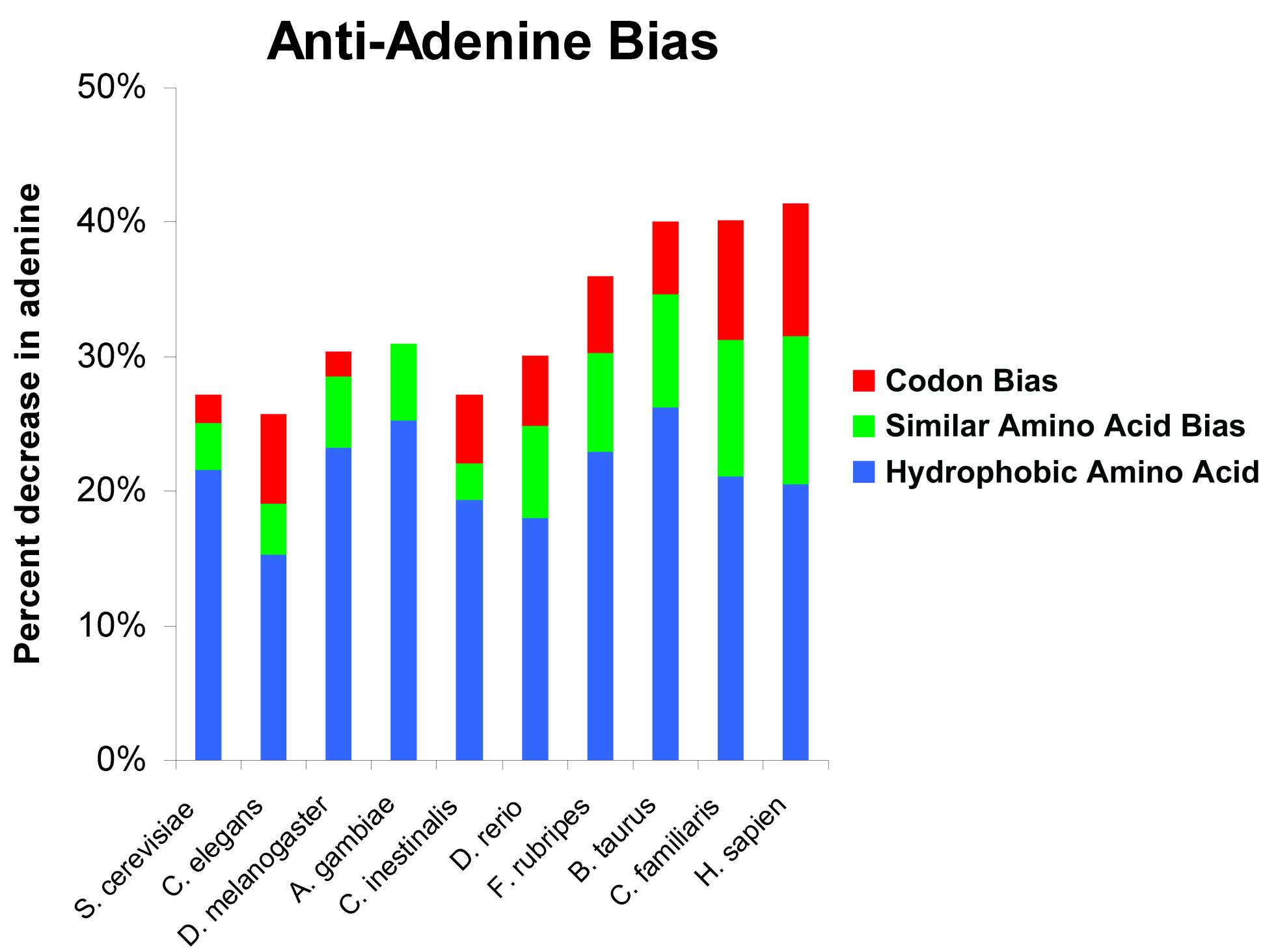

Anti-Adenine Bias
50%
40%
30%
20%
10%
0%
Percent decrease in adenine
S. cerevisiae
C. elegans
D. melanogaster
A. gambiae
C. inestinalis
D. rerio
F. rubripes
B. taurus
C. familiaris
H. sapien
Codon Bias
Similar Amino Acid Bias
Hydrophobic Amino Acid
