# Supplementary material for: The Signal Sequence Coding Region Promotes Nuclear Export of mRNA
Source: PLoS Biol. 2007 Dec 4;5(12):e322. doi: 10.1371/journal.pbio.0050322 (PMC2100149; doi:10.1371/journal.pbio.0050322)
Supplement: Supplementary file 2 [file pbio.0050322.sg001.doc]

Figure S1

t-Ftz-i

GGGAGACCCAAGCTTGTCGACGCCGCCACCATGGTACCGTGCACGCTGCTCCTGCTGTTGGCGGCCGCCCTGGCTCCGACTCAGACCCGCGCGACCATGGGGTGTTGTCCCGGCTGTTGTGACTACAAGGACGACGATGACAAAGGCAGGCTCGACTACTTGGACGTCTACTCGCCCCAGTCGCAGACGCAGAAGCTGAAGAATGGCGACTTTGCCACCCCTCCGCCAACCACGCCCACCTCTCTGCCGCCCCTCGAAGGCATCAGCACGCCACCCCAATCGCCGGGGGAGAAATCGTCGTCAGCTGTCAGCCAGGAGATCAATCATCGAATTGTGACAGCCCCGAATGGAGCCGGCGATTTCAATTGGTCGCACATCGAGGAGACTTTGGCATCAGGTAGGCATCACACACGATTAACAACCCCTAAAAATACACTTTGAAAATATTGAAAATATGTTTTTGTATACATTTTTGATATTTTCAAACAATACGCAGTTATAAAACTCATTAGCTAACCCATTTTTTCTTTGCTTATGCTTACAGATTGCAAAGACTCGAAACGCACCCGTCAGACGTACACCCGCTACCAGACCCTGGAGCTCGAAGGGTACCCATACGATGTTCCAGATTACGTCCTGCAGTAAGCTCGCTTTCTTGCTGTCCCAATTTCTATTAAACTCGAG
