# Supplementary material for: The Signal Sequence Coding Region Promotes Nuclear Export of mRNA
Source: PLoS Biol. 2007 Dec 4;5(12):e322. doi: 10.1371/journal.pbio.0050322 (PMC2100149; doi:10.1371/journal.pbio.0050322)
Supplement: Supplementary file 3 [file pbio.0050322.sg003.doc]

Figure S3

Name

Comment	SSCR Region, nucleotide/amino acid sequence

t-ftz	Translocated

ftz

AUGgUaccgUgcacgcUgcUccUgcUgUUggcggccgcccUggcUccgacUcagacccgcgcgaccAUG

containing the SSCR	M V P C T L L L L L A A A L A P T Q T R A T M

from the H2-K1 gene

c-ftz

cytoplasmic ftz

```
AUG
 M
```

3R-ftz	3 arginine mutations

```
AUGgUaccgUgcacgcUgcUccUgcGgUUgCGggccGCccUggcUccgacUcagacccgcgcgaccAUG
 M  V  P  C  T  L  L  L  R  L  R  A  R  L  A  P  T  Q  T  R  A  T  M
```

fs-ftz	Frame shift mutation

```
AUGgGUaccgUgcacgcUgcUccUgcUgUUggcggccgcccUggcUccgacUcagacccg-gcgaccAUG
 M  G  T  V  H  A  A  P  A  V  G  G  R  P  G  S  D  S  D  P   A  T  M
```

UUG-ftz	Start codon mutation

```
UUGgUaccgUgcacgcUgcUccUgcUgUUggcggccgcccUggcUccgacUcagacccgcgcgaccAUG
	  							       M
```

7A-ftz

7 silent A mutations

```
AUGgUaccAUgcacAcUgcUAcUgcUAUUggcAgccgcAcUAgcUccgacUcagacccgcgcgaccAUG
 M  V  P  C  T  L  L  L  L  L  A  A  A  L  A  P  T  Q  T  R  A  T  M
```

insulin	insulin SSCR

AUGGCCCUGUGGAUGCGCCUCCUGCCCCUGCUGGCGCUGCUGGCCCUCUGGGGACCUGACCCAGCCGCAGCC

ins-ftz	M A L W M R L L P L L A L L A L W G P D P A A A

5A-insulin	5 silent A mutations	AUGGC**A**CUGUGGAUGCGCCU**A**CUGCC**A**CUGCUGGC**A**CUGCUGGCCCU**A**UGGGGACCUGACCCAGCCGCAGCC

M A L W M R L L P L L A L L A L W G P D P A A A
